# Tap water as the source of a Legionnaires’ disease outbreak spread to several residential buildings and one hospital, Finland, 2020 to 2021

**DOI:** 10.2807/1560-7917.ES.2023.28.11.2200673

**Published:** 2023-03-16

**Authors:** Silja Mentula, Sohvi Kääriäinen, Sari Jaakola, Marjo Niittynen, Piia Airaksinen, Irma Koivula, Markku Lehtola, Ella Mauranen, Isto Mononen, Raija Savolainen, Susanna Haatainen, Outi Lyytikäinen

**Affiliations:** 1 Finnish National Institute for Health and Welfare, Helsinki, Finland; 2 ECDC Fellowship Programme, Field Epidemiology path (EPIET), European Centre for Disease Prevention and Control (ECDC), Solna, Sweden; 3 Environmental Health, City of Kuopio, Finland; 4 Kuopio University Hospital, Kuopio, Finland; 5 Kuopion Vesi, Kuopio, Finland; 6 Health Services, City of Kuopio, Finland; 7 Real Estate Services, City of Kuopio, Finland

**Keywords:** Legionella, outbreak, water network, whole-genome sequencing

## Abstract

In Finland, all microbiology laboratories notify *Legionella* findings and physicians notify Legionnaires’ disease (LD) cases to the National Infectious Disease Register. All cases are interviewed, and water samples obtained from potential places of exposure. *Legionella* isolates from humans and water are compared by whole genome sequencing (WGS). In March 2021, *Legionella pneumophila* serogroup 1 (Lp 1) pneumonia cases increased in one Finnish city (120,000 inhabitants) where single LD cases are detected annually. We identified 12 LD cases, nine living in different residential buildings and three nosocomial, linked by identical human and/or water isolates. Three of these cases were from January 2020, October 2020 and February 2021 and identified retrospectively. Eleven were diagnosed by urinary antigen test, 10 by PCR and five by culture; age ranged between 52 and 85 years, and 10 had underlying diseases. Nine of 12 homes of LD cases and 15 of 26 water samples from the hospital were positive for Lp 1, with concentrations up to 640,000 cfu/L. Water samples from regional storage tanks were negative. Positivity in homes and the hospital suggested inadequate maintenance measures. Enhanced surveillance combined with WGS was crucial in detecting this unusual LD outbreak related to domestic and hospital water systems.

**Key public health message**

**What did you want to address in this study?**

Legionellae are bacteria found in fresh water and soil. They can grow in man-made water systems and cause pneumonia by inhalation of aerosolised water. Showers, air conditioning, cooling towers, whirlpool spas and decorative fountains are common sources for transmission. When pneumonia caused by *Legionella pneumophila* serogroup 1 increased in spring 2021, we aimed to find the source of the outbreak to implement control measures and prevent further cases.

**What have we learnt from this study?**

We interviewed *Legionella* pneumonia cases and collected water samples from places where the cases may have been exposed. The genomic sequences of *Legionella* isolates were compared to identify the environmental source of the infections and find out if the cases were linked to each other. *Legionella* isolates from humans were similar, even though the cases had been exposed in different residential buildings and in one hospital. These places were served by a common water network.

**What are the implications of your findings for public health?**

Surveillance including case interviews, environmental sampling and sequencing is important in the detection of *Legionella* pneumonia outbreaks. This is especially true for the most important *Legionella* type, *Legionella pneumophila* serogroup 1, which can be diagnosed by urine antigen test without a bacterial culture and isolate.

## Background

Legionnaires’ disease (LD) is an important cause of atypical pneumonia and can be community-acquired, travel-associated or nosocomial [1-3]. Besides age and having a weak immune system, or a chronic lung disease, former and current smokers are at increased risk for LD [3]. Another risk factor is a stay at a hotel or similar accommodation [1,2]. Case fatality is 8–12%, being higher in elderly people, those with underlying diseases and nosocomial cases [3]. Legionnaires’ disease is caused by Gram-negative aerobic *Legionella* bacteria that are frequent in fresh water and soil. *Legionella* can enrich in man-made water systems, especially in stagnant water in temperatures between 20 °C and 45 °C. *Legionella* is not an exceptional finding in residential water systems and can cause sporadic infections associated with non-hospital facilities [4,5]. However, large LD outbreaks have often been caused by single or multiple cooling towers [1]. Transmission occurs mainly by inhalation of aerosols or aspiration of water containing *Legionella*.

Among the 30 pathogenic *Legionella* species, *Legionella pneumophila* serogroup 1 (Lp 1) is responsible for the majority of LD cases in Europe [2]. In nosocomial cases, other serogroups and species are common as well. The diagnosis of LD is based on urinary antigen test (UAG), PCR and/or culture from respiratory specimens or serology. Most UAG tests are specific only for Lp 1, thus the detection of other serogroups or species requires PCR and/or culture. Diagnosis of LD should prompt to identify the source of infection and trace other cases, as there is the potential of an outbreak [1].

In Finland, the annual number of LD cases ranged between five and 44 in the period from 2010 to 2020, which corresponds to an incidence of 0.8 per 100,000 population, lower than the average incidence in European countries of 2.2 per 100,000 in 2019 (range by country: 0.1–9.4/100,000) [6]. More than half of the Finnish cases were linked to travelling abroad. No major LD outbreaks occurred, except some small clusters, including one nosocomial outbreak [7] and two industrial wastewater-associated cases [8]. Since 2014, enhanced surveillance has been conducted by interviewing all LD cases to identify the potential places of exposure, collecting environmental samples and, since 2016, comparing human and environmental isolates by whole genome sequencing (WGS) in the reference laboratory in the Finnish Institute for Health and Welfare (THL).

## Outbreak detection

**Figure 1:**
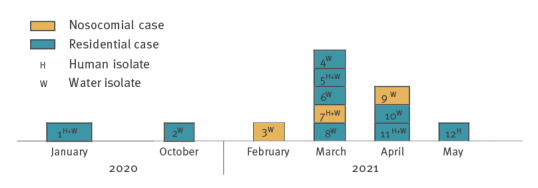
Monthly number of Legionnaires’ disease cases by date of diagnostic test, city in Northern Savonia healthcare district, Finland, January 2020–May 2021 (n = 12)

The outbreak investigation was initiated in March 2021 when we detected five LD cases within one month in a Finnish city with 120,000 inhabitants in the Northern Savonia healthcare district (247,000 inhabitants), where previously between one and three LD cases have been detected annually. Four more cases appeared during April and May 2021, and further cases from January 2020, October 2020 and February 2021 linked to this outbreak were detected retrospectively (Figure 1). Five of the cases were from the same residential area, living in different buildings on different streets. There were also cases living in three neighbouring residential areas (n = 4) and in one local hospital (n = 3). The residential areas and the hospital were located within a maximum distance of 8 km from each other and 4–8 km from the water plant which provided water for the area of the community. The residential buildings were low-rise apartments or terraced houses built in the late 1980s and early 1990s. The hospital buildings were built between 1914 and 1990. All residential buildings and the hospital had a central heating system.

Case numbers with a human isolate available for typing are marked with H and cases with water isolate available for typing with W.

The objectives of the outbreak investigation were to detect the source of the outbreak, to control the outbreak and to report this unusual LD outbreak in one Finnish city with 12 cases extending over 1 year, January 2020 to May 2021.

## Methods

### Surveillance and epidemiological investigation

Finnish microbiology laboratories notify all *Legionella* sp. findings, and physicians notify LD cases to the National Infectious Disease Register. Notifications include demographic data, date and type of specimen, laboratory method and preceding travel history. Legionnaires’ disease cases are also under enhanced surveillance: all cases are routinely interviewed using a structured form (underlying diseases, smoking, travel/hospital history, aerosol exposures at work and leisure time) to identify the possible places of exposure within the incubation period (2–10 days) [2] and based on these data, water or soil samples are collected from homes and/or other potential places of exposure. Human and environmental isolates are compared by WGS at THL.

### Case definitions

We defined an LD case as a patient with pneumonia and a specimen positive for Lp 1 in UAG, PCR or culture from a respiratory specimen. An LD case was classified as residential when there was no hospital history or as nosocomial when a patient had stayed in the hospital during the incubation period. The classification was supported by environmental findings and inclusion to the outbreak was confirmed by detection of the Lp 1 outbreak genotype from a human and/or water isolate in the city in Northern Savonia healthcare district in 2020 or 2021.

### Environmental and microbiological investigation

As part of the enhanced surveillance, tap water samples were collected in a similar manner from the cases’ homes, including hot water samples from shower heads, cold water samples from shower taps and hot water from kitchen taps, mostly within 2–3 weeks of the LD diagnosis (Table 1). In two residential buildings, water samples were also collected from the shared sauna facilities. In the hospital, sampling was first conducted on the wards where the nosocomial LD cases had stayed, and later on other wards as a precaution. Samples were also taken from a hotel and an industrial site where two residential LD cases had worked, and in one nursing home where one nosocomial LD case had stayed before their hospitalisation. Samples were taken first without running the water and water temperatures were measured at three time points: at the start, 1 min and 2−3 min after opening the tap.

**Table 1:** Number and timing of water samples taken in each sampling site and the link to the Legionnaires’ disease cases in city in Northern Savonia healthcare district, Finland, January 2020–May 2021 (n = 90)

Case	Sampling site^a^	Date of diagnostic test	Initial sampling round	Number of water samples		Number of control rounds	Total number of samples in control rounds		Last control round
				Hot	Cold		Hot	Cold	
1	Home	21 Jan 2020	4 Feb 2020	2	1	2	5	3	11 Mar 2021
2	Home	22 Oct 2020	18 Nov 2020	3	2	4	5	5	11 Feb 2021
3	Home	23 Feb 2021	18 Mar 2021	2	1	0	Not sampled		
	Nursing home	23 Feb 2021	18 Mar 2021	2	1	0	Not sampled		
	Hospital ward A	23 Feb 2021	23 Mar 2021	3	1	4	7	1	13 Oct 2021
4	Home	6 Mar 2021	30 Mar 2021	2	1	1	2	1	4 May 2021
	Industrial site	6 Mar 2021	30 Mar 2021	3	4	0	Not sampled		
5	Home	19 Mar 2021	30 Mar 2021	2	1	1	2	1	20 Apr 2021
6	Home	22 Mar 2021	7 Apr 2021	4	2	2	4	5	2 Jul 2021
7	Home	23 Mar 2021	20 Apr 2021	2	1	0	Not sampled		
	Hospital ward B	23 Mar 2021	13 Apr 2021	3	2	2	3	1	13 Oct 2021
8	Home	25 Mar 2021	7 Apr 2021	2	1	0	Not sampled		
9	Home	5 Apr 2021	10 Jun 2021	2	1	0	Not sampled		
	Hospital ward C	5 Apr 2021	13 Apr 2021	5	3	2	3	3	24 May 2021
10	Home	13 Apr 2021	4 May 2021	2	1	1	2	0	2 Jun 2021
11	Home	29 Apr 2021	2 Jun 2021	2	1	0	Not sampled		
	Hotel	29 Apr 2021	19 May 2021	5	5	2	6	2	18 Jun 2021
12	Home	1 May 2021	20 May 2021	2	1	0	Not sampled		
Hospital, other wards^b^		NA	6 May 2021	7	2	3	15	1	13 Oct 2021
Regional storage tanks		NA	10 May 2021, 19 May 2021	0	3	0	Not sampled		
**Total**				**55**	**35**	**24**	**54**	**23**	**NA**

NA: not applicable.

^a^ Home: hot water from shower head and cold water from shower tap, hot water from kitchen tap, additionally cold water from sink taps of the shared sauna facilities (Cases 2 and 6). Hospital, nursing home and hotel: hot water from shower head and cold water from shower tap, hot and cold water from sink tap and hot water from bidet shower when applicable. Industrial site: hot water from shower head and cold water from shower tap, hot and cold water from sink and kitchen taps, cold water from a hose.

^b^ Sampling in other wards was precautionary, not related to the detected cases.

Approximately 2–4 weeks after the implementation of control measures, control samples were collected from all sites and sampling points where *Legionella* concentrations exceeded 1,000 colony forming units per litre (cfu/L) [9]. One municipal water company provided water for the area of the city from a single water plant which is located close to the city centre. We took water samples from the regional storage tanks.

#### Human samples

Patient specimens were examined in the local clinical microbiology laboratory. Decision on the diagnostic method (UAG, PCR and/or culture) was clinical.

#### Water samples

Water samples were collected by local health inspectors or by THL water microbiology laboratory personnel as part of the enhanced surveillance. Samples were analysed at THL by culture according to the SFS-EN ISO 11731:2017 standard [10].

#### Genotyping

Serotyping of human isolates and all genotyping was performed in the reference laboratory at THL. Human and water isolates were compared by WGS core genome multilocus sequence typing (cgMLST). The WGS was performed on a MiSeq instrument (Illumina, San Diego, United States (US)). Library preparation was done with NexteraXT V2 DNA sample preparation kit (Illumina). The cgMLST was performed using SeqSphere cgMLST tool version 8.2.0 (Ridom GmbH). The Sequence Based Typing protocol for *Legionella* (SBT) profile was checked with the legsta tool from GitHub [11].

#### Statistical analysis

We compared proportions for categorical variables by chi-squared test and *Legionella* concentrations by quantile regression with fixed effects [12]. The analyses were performed using Stata version 17.0 (StataCorp LLC, College Station, US).

## Results

We identified 12 LD cases; nine were residential and three nosocomial. Four were in working life and none had travelled. The median age was 65 years (range: 52–85), seven were female and five were male, and 10 of the 12 were taking immunosuppressive medication and/or had underlying disease. One case was known to be a smoker. All residential cases were hospitalised for LD, one of the nosocomial cases died. The LD was diagnosed by UAG in 10 cases, PCR in nine and bacterial culture in five; all PCR tests and cultures were positive for Lp 1.

### Typing

Genotyping was performed for all five available human isolates and for selected water isolates, representing both hot and cold water from eight of nine homes (16 isolates) and from the hospital (six isolates) (Figure 1). One stored water isolate of Lp 1 obtained from a home was no longer viable, but the human isolate for the corresponding case was available (Case 12). The first and third case were identified when we compared the outbreak strains with earlier *L. pneumophila* isolates in the THL cgMLST library and discovered one identical human and one identical water isolate from the city in January 2020 (Case 1) and February 2021 (Case 3). We then sequenced one water isolate connected to an LD case in October 2020 diagnosed by UAG and found it to be identical (Case 2). In 2020, there had been altogether three LD cases in the city, two of them caused by Lp 1 and the third by *L. longbeachae*.

**Figure 2:**
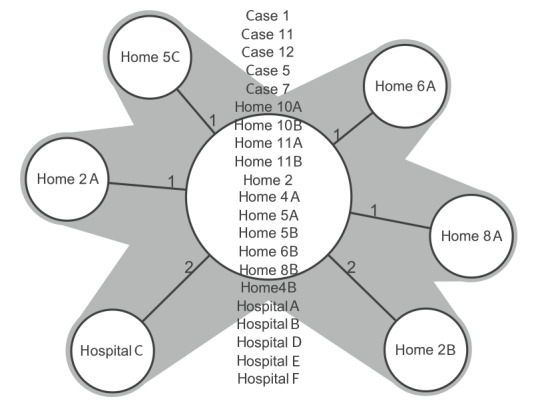
Minimum spanning tree of the *Legionella pneumophila* isolates by Ridom SeqSphere cgMLST, city in Northern Savonia healthcare district, Finland, January 2020–May 2021 (n = 27)

All 27 isolates were identical or with minor differences (maximum 2/1,443 targets) (Figure 2). The mean percentage of good targets was 99.4% (range: 97.8−99.9%) and the mean average coverage was 151 (range: 90−262). The isolates were complex type 100. The legsta SBT tool generated a similar profile for all isolates for genes *flaA*, *pilE*, *asd*, *mip*, *mompS*, *proA*, *neuA* as 3, 4, 1, ND, 14, 9, 11. The sequence type could not be named because the *mip* allele number was missing. The LD cases’ link to the outbreak was confirmed by water isolate (seven cases), human isolate (five cases) or both (four cases).

cgMLST: core genome multilocus sequence typing.

*L. pneumophila* 1443/1470 cgMLST v1.1. *L. pneumophila* cgMLST complex type/cluster-alert distance: 4. A 1–2 allele difference was detected in six isolates (outside circles), while 21 isolates were 100% identical (middle circle). Isolates marked ‘Case’ are human isolates, all others are water isolates from the hospital and homes.

### Environmental investigation

#### Water samples

We collected 90 water samples (35 cold and 55 hot water) from 20 sites during the initial sampling round (Table 1). *Legionella* genus was found in 56 of 90 samples from 13 of 20 sites, including nine homes, several wards in the hospital and the hotel. Serogroup Lp 1 was found in nine of 12 homes of LD cases (residential Cases 1–2, 4–6, 8, 10–12) and in the hospital (nosocomial Cases 3, 7, 9) (Table 2). In three homes, we detected only Lp 1, in six homes Lp 1 and other *Legionella* species, in the hospital Lp 1, Lp 2−14 and other *Legionella*, and in the hotel Lp 2−14 and other *Legionella*. Water samples from the nosocomial cases’ homes were negative for *Legionella*. Water samples from the hotel did not grow Lp 1, and samples from the industrial site and nursing home were negative for *Legionella*. In five of nine homes (residential Cases 1, 2, 4, 5 and 10), the Lp 1 concentration was ≥ 1,000 cfu/L and in four of nine homes (residential Cases 6, 8, 11 and 12) it was ≤ 500 cfu/L. In the hospital, an Lp 1 concentration > 1,000 cfu/L was detected three times.

**Table 2:** Diagnostic tests of 12 Legionnaires’ disease cases, highest *Legionella* concentrations in water samples, lowest hot water and highest cold water temperature during the initial sampling, city in Northern Savonia healthcare district, Finland, January 2020–May 2021 (n = 90)

Case, N/R	Diagnostic test	Water samples from home				Water samples outside home			
		Lp 1 (cfu/L)	Non-Lp 1 (cfu/L)	Hot water (lowest °C)	Cold water (highest °C)	Lp 1 (cfu/L)	Non-Lp 1 (cfu/L)	Hot water (lowest °C)	Cold water (highest °C)
1, R	PCR, culture	15,000	0	49.8	11.6	Not sampled			
2, R	UAG	20,000	30,000	50.7	18.2	Not sampled			
3, N^a,b^	UAG	0	0	50.3	8.0	3,400	200	50.7	11.4
						0	0	57.2	13.8
4, R^c^	UAG, PCR	1,000	5,500	51.7	9.7	0	0	53.1	19.2
5, R	UAG, PCR, culture	1,800	0	43.1	11.3	Not sampled			
6, R	UAG	500	70,000	52.8	14.0	Not sampled			
7, N^a^	UAG, PCR, culture	0	0	57.2	6.7	640,000	0	50.9	9.0
8, R	PCR	250	250	52.7	8.6	Not sampled			
9, N^a^	UAG, PCR	0	0	44.6	22.2	20,000	0	47.2	7.9
10, R	UAG, PCR	1,500	25	47.4	14.3	Not sampled			
11, R^d^	UAG, PCR, culture	100	0	55.0	13.1	0	180,000	47.7	9.0
12, R	UAG, PCR, culture	50	550	51.6	12.8	Not sampled			

cfu: colony-forming unit; Lp 1: *Legionella pneumophila* serogroup 1; N: nosocomial; R: residential; UAG: urinary antigen test.

^a^ Sample outside the home taken at the hospital.

^b^ Sample outside the home taken at the nursing home.

^c^ Sample outside the home taken at the working place (industrial site).

^d^ Sample outside the home taken at the working place (hotel).

Temperature at 1 min after the opening the tap. The results for the hospital are from the three wards where the nosocomial cases stayed. ‘Non-Lp 1’ includes *L. pneumophila* serogroups 2–14 and other *Legionella* species.

Among all samples positive for *Legionella* genus, the concentrations ranged between 5 and 640,000 cfu/L (median: 1,500 cfu/L). It was > 1,000 cfu/L in 30 of 56 (54%) samples and ≤ 500 cfu/L in 21 of 56 (38%). The concentrations did not differ significantly between Lp 1 (n = 42) and non-Lp 1 *Legionella* (n = 21) (median: 620 vs 3,500 cfu/L; p = 0.931). Hot water samples were not more often *Legionella*-positive than cold water samples (34/46 vs 22/33; p = 0.484), and *Legionella* concentrations did not differ between hot and cold water (median: 1,650 vs 570 cfu/L; p = 0.285).

Hot water temperatures (1 min) were below 50 °C at one or two of the sample points in four homes (Cases 1, 5, 9 and 10), at one sample point in the hospital and at two sample points in the hotel (Table 2). Cold water temperature was above 20 °C at a single sample point in one home (Case 9). The mean hot water temperatures were slightly higher at *Legionella*-negative than *Legionella*-positive sample points (53.1 °C vs 51.2 °C, 1 min). The mean cold water temperature was higher at *Legionella*-negative than -positive sites (13.7 °C vs 10.7 °C, 1 min). The lowest hot water temperature (44.6 °C, 1 min) and highest cold water temperature (22.2 °C, 1 min) were both measured from a *Legionella-*negative site.

#### Water company

One municipal water company provided water for the city from a single water plant which was located close to the city centre. Water was transferred via local storage tanks and pumping stations. Tanks were washed and disinfected every 4 to 5 years. There was no regular *Legionella* monitoring, but samples were taken from the regional pumping station and mains as part of a research project in April 2021 and were negative for *Legionella*. Samples collected in connection with the outbreak in May 2021 from regional storage tanks were also negative for *Legionella*.

## Outbreak control measures

We implemented control measures right after the confirmation of *Legionella* in each site. Immediate control measures included flushing each tap, heat shock treatment and/or increasing hot water temperatures and changing of water mixers, shower heads and hoses if they were in poor condition. Point-of-use microbiological grade filters were installed in the hospital.

Altogether 76 control samples were collected from sites where *Legionella* concentrations exceeded 1,000 cfu/L: six of nine *Legionella*-positive homes (34 samples), the hospital (34 samples) and the hotel (eight samples). The hot water temperatures at *Legionella*-positive sites increased by each control round from a mean of 52.8 to 59.6 °C (Table 3). *Legionella* concentrations decreased below the detection limit in three homes by the first control round, in two homes and in the hotel by the second. In one building, the counts in the apartment dropped by the first control round but the shared sauna remained positive until disinfection measures and four rounds of control sampling. Disinfection (chlorination) was performed in two residential buildings where the *Legionella* concentration remained > 1,000 cfu/L in cold water control samples. Surface water with free chlorine treatment had been used in the water company, however, it was known that the chlorine levels were low at the distal sites in residential areas several kilometres away from the plant. A change to UV treatment combined with monochloramine treatment had already been planned and was carried out in summer 2021.

**Table 3:** Water samples during the initial sampling for *Legionella* and four control rounds, city in Northern Savonia healthcare district, Finland, February 2020–October 2021 (n = 166)

Sampling round	Hot water					Cold water				
	Sites	Sample points	Temperature	*Legionella*-positive	*Legionella *>1,000 cfu/L	Sites	Sample points	Temperature	*Legionella*-positive	*Legionella *>1,000 cfu/L
	n	n	Mean °C (range)	n	n	n	n	Mean °C (range)	n	n
Initial	11	55	52.8 (44.6–60.8)	37	24	11	35	11.2 (5.5–22.2)	20	10
1st control	8	36	57.5 (42.1–62.6)	14	8	7	13	11.1 (7.9–15.7)	10	4
2nd control	5	8	58.0 (53.4–64.3)	4	6	4	6	14.1 (10.2–17.8)	2	2
3rd control	2	7	59.1 (56.8–62.0)	1	0	2	2	14.4 (12.1–16.7)	1	1
4th control	1	3	59.6 (58.0–59.8)	0	NA	1	1	10.8	0	NA

Cfu: colony-forming units; NA: not applicable.

Mean water temperatures (1 min), number of *Legionella-*positive samples, and number of samples where concentration was over 1,000 cfu/L are presented in all sample points (taps) for *Legionella*-positive sites (nine homes, hospital and hotel).

To monitor the level of contamination and the effects of control measures in the hospital, we conducted five additional sampling rounds to cover the three wards with cases and also other wards located in different buildings, including 41 sample points with altogether 60 samples with two to four control rounds per ward. Hot water temperatures varied widely between the different sample points in the first sampling round (mean: 54 °C; range: 47.2−60.2 °C; 1 min), after which temperatures increased (mean: 57.3 °C; range: 49.4−62.4 °C; 1 min), and finally no *Legionella* was found. The decision to install a chlorination system was made to prevent future contamination.

Permanent recommendations included a request for regular flushing, elevating hot water temperatures above 55 °C, and overall enhanced maintenance and monitoring of the water systems, especially in high-risk buildings, such as healthcare settings.

We monitored the effect of flushing on temperatures by using all obtained water temperature measurements, including 305 hot water measurements (103 sample points) and 158 cold water measurements (54 sample points). The effect of flushing on temperatures was clear, the mean temperatures dropped 6.1 °C (from 15.4 to 9.3 °C) for cold water and increased 5.8 °C (from 50.7 to 56.5 °C) for hot water by flushing 2−3 min.

## Discussion

We described an LD outbreak in a Finnish city during 2020 and 2021 with few community-acquired cases per year in the past. All LD cases were exposed in different buildings or hospital wards but were linked to each other by time and the common water system. The outbreak was confirmed by WGS, all human and water Lp 1 isolates were identical or very closely related. Enhanced surveillance by interviewing all LD cases and environmental sampling of potential places of exposure was crucial in the detection of this outbreak. As most LD cases were diagnosed by UAG or PCR instead of culture, human isolates were not available for all cases for typing and comparison. However, water isolates from the places of exposure were used to link the cases to the outbreak.

The outbreak extended to nine residential buildings and one hospital located in five different parts of the city, served by a common water network. Even though the bacteria were most probably initially spreading through the city water network, the cases were limited to certain buildings where the same *Legionella* strain was enriched. The most important contributing factors were probably inadequate maintenance measures by users, such as low water consumption in the buildings involved, as well as the vulnerability of the LD cases. We only investigated sites associated with the LD cases, and as the source of infection was found to be the water system (showers) associated with each case, there was no immediate cause for further sampling. Thus, the extent of contamination in other buildings of the residential areas with cases, in cooling towers and in other parts of the city remain unknown. However, more cases were sought by alerting healthcare professionals and giving public announcements of the outbreak. Typically for *Legionella* outbreaks [1], most exposed did not get infected. The cases shared risk factors including age and underlying medical conditions. Most had also some physical limitations and reported a relatively low water consumption enabling stagnant water in the taps, hoses and pipes in the apartments. Showering was the most likely route of infection for all cases. Similar findings pointing out the relevance of host risk factors, with only single or few cases among many exposed residents, have been reported previously [5,13]. However, our outbreak is exceptional as we had a matching isolate among seemingly sporadic cases and in the hospital cluster.

Interestingly, all residential buildings were equally old. Four of them were owned by a single municipal public housing company and all were maintained by private maintenance and service companies. The buildings were typical low-rise buildings in that area with no special features explaining the outbreak. Control measures were implemented rapidly in each building. The maintenance service in the affected hospital got support from another hospital with a history and experience of controlling *L. pneumophila* serogroup 5 [14]. By implementing the new European Union Drinking Water Directive (2020/2184), which mandates regular *Legionella* monitoring in high-risk buildings, defined by municipalities in Finland, healthcare-associated infections could be prevented [15].

*Legionella* concentrations were < 1,000 cfu/L in three homes (Lp 1: 50, 100 and 250 cfu/L), suggesting that also lower *Legionella* concentrations can be infectious. However, infectivity may also be affected by other factors, such as exposure time, strain virulence and host susceptibility. The concentrations may vary over time. Here, the interval between the time of diagnosis and sampling varied from 1 to 5 weeks. The majority of the contaminated water systems yielded multiple *Legionella* species; among them, the concentrations of Lp 1 were often lower than those of other *Legionella*. Still, all human isolates were Lp 1, which is in line with *L. pneumophila* being the most virulent *Legionella* species [16]. It is also noteworthy that not all samples from the identified sources of infection yielded Lp 1. Thus, it was essential that multiple sample points were initially tested and multiple isolates serotyped. In an Italian study, 22% of the hot domestic water samples were *Legionella*-positive with a 75% rate of positivity for *L. pneumophila* [17]. In Canada, 33% of domestic water systems among community-acquired LD cases were *Legionella*-positive but only 14% of the findings matched with human isolates by genotyping [5]. In Finland, the most likely source of infection was identified by environmental sampling for 50–60% of domestic *Legionella* cases during the last decade, but the patient isolate was available for only around 20% of the cases.

The mean water temperatures were largely similar in *Legionella*-positive and -negative sites. Only few samples were outside the temperature recommendations for hot and cold water, suggesting that the technical settings, house pipelines, thermal insulation and the adjusted level of heating were not the main cause of the outbreak. This is further supported by the fact that there were only single cases in each building. We think that the regular use of plumbing fixtures or any water containing equipment is equally important as the upkeeping of the recommended temperatures to control *Legionella.*

Recommendations for hot water in Finland are > 50 °C for buildings built before 2007 and > 55 °C for those built or renovated after 2007. We isolated *Legionella* despite temperatures > 50 °C, thus higher temperatures (even 60–65 °C) may be needed, especially knowing that *Legionella* is able to grow inside thermotolerant amoebae [9]. *Legionella* was also found in lower concentrations in cold water taps with temperatures where *Legionella* should not grow but can remain viable. The initial control measures were effective only in three homes; at all other sites, more control measures and further sampling were needed. Two sites required disinfection using biocides because *Legionella* persisted in cold water. In Germany, there was no clear correlation between cold water temperature and *Legionella* contamination rate in healthcare facilities [18]. A high positivity rate (35%) among cold water samples < 20 °C suggested that no temperature threshold can be defined below which cold water would be considered free of *Legionella*.

We found some exceptionally high *Legionella* concentrations. When a water system is heavily contaminated, a total eradication of *Legionella* is seldom possible but lowering the concentration can be achieved by continuous technical control measures [9]. A system may be colonised by a long- term predominant strain causing LD cases infrequently, while the sporadic strains are of less pathogenetic relevance [19]. In France, a long-term Lp 1 colonisation of the city water network was reported, but unlike in our situation, the particular clone was not connected to LD cases [20]. Long-term colonisation has been reported especially for hospital buildings but also for municipal water systems [19-22].

The water temperatures would have favoured the growth of *Legionella* in the apartment of one of the nosocomial cases. However, all samples were negative, confirming that the water mains had not been heavily contaminated. There was also no known incident in the water plant explaining any sudden increase in the nutrients, decrease in disinfection efficiency or any other change in the process that would cause the increase in *Legionella*. As reported earlier, a change in primary disinfectant can cause an interruption in the corrosion control, a decrease in disinfectant residuals and an increase in lead in the distribution system, and create favourable conditions for *Legionella* [23]. Similarly, *Legionella* clusters have been connected to a switch in raw water source from non-corrosive water to corrosive river water [24]. Coronavirus disease (COVID-19) pandemic closures have been shown to affect water consumption [25]. In the concerned city, water usage decreased by 2% in the city centre and increased by 2% in the residential areas between March and December 2020.

*Legionella* concentrations did not always decrease immediately after the control measures, highlighting the difficulties in defining the appropriate number and level of actions and the need for control samples. The mean hot water temperatures rose with the sequential sampling rounds. If taps are not used regularly, the water gradually reaches room temperature. As shown in our study, water temperature changed by 4 °C already after 1 min of flushing and by 6 °C after 2–3 min of flushing, thereby improving water quality by removing residual water from the pipes. Actions need to be tailored to a site’s specific situation, while considering safety issues such as the risk of skin burns during heat shock.

To ensure proper management, guidance and supervision of the investigation and control measures, the outbreak was handled by a multi-professional working group consisting of technical, environmental health and clinical experts from all parties involved, including THL, the city environmental and health authorities, the water company, the regional university hospital, support services and the housing company. The city published two press releases, one on the cases and the environmental findings and the other on prevention methods and recommendations for all residential buildings. Local healthcare professionals were informed about the *Legionella* outbreak and advised to test for *Legionella* with a low threshold.

## Conclusions

Enhanced surveillance including water samples for single cases combined with WGS for isolates was crucial in detecting and defining this unusual LD outbreak related to the city water network. Inadequate maintenance measures and probably low water consumption, together with the vulnerability of the cases, contributed to the outbreak.
